# Physical Activity, Sedentary Behavior, Anxiety, and Pain Among Musicians in the United Kingdom

**DOI:** 10.3389/fpsyg.2020.560026

**Published:** 2020-12-03

**Authors:** Raluca Matei, Jane Ginsborg

**Affiliations:** ^1^Centre for Sustainable Working Life, Department of Organizational Psychology, Birkbeck, University of London, London, United Kingdom; ^2^Centre for Music Performance Research, Royal Northern College of Music, Manchester, United Kingdom

**Keywords:** physical activity, conservatoire students, sedentary behavior, behavior change, anxiety, PRMDs, conservatoire training, health education

## Abstract

**Context and Aims:**

Although some exercise-based interventions have been associated with lower levels of pain and performance-related musculoskeletal disorders (PRMDs) among musicians, the evidence is still mixed. Furthermore, little is known about musicians’ general engagement in physical activity (PA), their knowledge of PA guidelines, or the relevant training they receive on pain prevention and the sources of such training. Similarly, little is known about the relationship between PA and PRMDs and other risk factors for PRMDs.

**Methods:**

Following a cross-sectional correlational study design, both standardized and *ad hoc* measurements were used to investigate self-reported PA [International Physical Activity Questionnaire – Short Form (IPAQ-SF)], knowledge of PA guidelines, and barriers to engaging in PA [Centers for Disease Control (CDC); Determinants of Physical Activity Questionnaire (DPAQ)]; sedentary behavior [Sedentary Behavior Questionnaire (SBQ)]; pain [36-Item Short Form Survey Instrument (SF-36)] and PRMDs (frequency and severity); reported physical exertion (RPE); anxiety [Hospital Anxiety and Depression Scale (HADS)]; practice behaviors (e.g., practice time; taking breaks frequency; warming up); and relevant training among conservatoire students in the United Kingdom. The entire set of questionnaires was administered both online and via hard copies between June 2017 and April 2018.

**Results:**

Demographic information was obtained from 111 respondents, mostly undergraduate students (UGs) from seven conservatoires. They reported high levels of engagement in PA, despite poor knowledge of PA guidelines. Teachers were the most frequently mentioned source of pain prevention information (by 43% of respondents), and 62% agreed that they had received advice on why they should engage in cardio PA. Sedentary behavior was comparable to normative data. Levels of bodily pain and PRMDs were low, but 43% showed “abnormal” clinical anxiety and found playing their instruments “somewhat hard” (RPE) on average. Bodily pain interfering with practice and performance was positively correlated with frequency and severity of PRMDs, anxiety, and RPE. Frequency and severity of PRMDs were also associated with sedentary behavior at the weekend. Anxiety was associated with RPE. No association was found between PA and PRMDs.

**Conclusion:**

The relationship between PA and PRMDs and pain remains unclear and needs further investigation. While health education needs to be improved, other pathways may need to be taken. Given the high levels of anxiety, the ideology of Western classical music itself may need to be challenged.

## Introduction

### Physical Activity, PRMDs, and Anxiety

The World Health Organization recommends that adults aged 18–64 years should engage in at least 150 minutes of moderate-intensity aerobic physical activity (PA) per week, or 75 min of vigorous-intensity PA. Muscle-strengthening activities are also recommended on two or more days per week (World Health Organization [WHO], 2010). The same guidelines are endorsed by the UK Department of Health and Social Care (Department of Health and Social Care [DHSC], 2019). Activities that count as moderate aerobic activity include brisk walking, riding a bicycle, hiking, volleyball, and pushing a lawn mower. Vigorous-intensity PA includes jogging, fast swimming, football, aerobics, gymnastics, and martial arts. Finally, lifting weights, working with resistance bands, push-ups and sit-ups, yoga and Pilates count as activities that help strengthen muscles (National Health Services [NHS], n.d.).

Several interventions aimed at preventing or mitigating musicians’ performance- or playing-related musculoskeletal disorders (PRMDs) have, to date, used various forms of PA such as general fitness, muscle strengthening, and endurance (Ackermann et al., 2002; de Greef et al., 2003; Kava et al., 2010; Chan et al., 2014a,b; Nygaard Andersen et al., 2017; Lundborg and Grooten, 2018; Roos and Roy, 2018). It should be noted that in these studies control groups were not always recruited; participants were not always randomized; some of the interventions were of short duration; in some instances, adherence was low; and interventions were sometimes so complex that it was impossible to isolate their key ingredients. Notwithstanding these limitations, the results of the studies showed associations between participation in the intervention program and reduced frequency and severity of PRMDs, pain, and perceived exertion; and improvements in objective measurements including isometric strength and endurance (Matei, 2019).

Most recently, the authors of the first study to investigate the self-reported PA and objective fitness levels of 483 musicians, for the most part students at UK conservatoires, concluded that while the majority exceeded the minimum weekly amount of PA, which was positively correlated with performance on tests of lung function, sit and reach, press-up and cardiovascular fitness, their performance on the plank, press-up, and sit and reach tests was poor in comparison with that reported for the population in the same age range (Araújo et al., 2020; Healthy Conservatoires, n.d.).

Physical activity has been associated with lower levels of anxiety in the general population (Conn, 2010; Rebar et al., 2015) and reductions in anxiety, via complex physiological mechanisms, in non-clinical adult populations, according to meta-meta-analytic findings, although effect sizes are small (Anderson and Shivakumar, 2013; Rebar et al., 2015). Musicians who report being physically active have lower levels of MPA than those who are “inactive” (Rocha et al., 2014). They also exhibit less anxiety after giving musical performances (Wasley et al., 2012). Regular PA has also been associated with lower perceived exertion during rehearsals (Wilke et al., 2011). However, there are currently no data on the relationship between musicians’ PA and (non-performance) anxiety. There could be a link between anxiety and PRMDs because muscular tension resulting from anxiety increases the risk of physical injury (Kava et al., 2010). While PRMDs could be caused by the somatization of psychological distress, the findings to date are based on correlational data so firm conclusions cannot be drawn (Ackermann et al., 2014). The present study explores anxiety in general rather than focusing on music performanc anxiety in particular, in an attempt to capture clinically meaningful results that might be applicable not only to music performance but more widely.

### Knowledge of Physical Activity

Although the evidence for the association between awareness of guidelines for PA and actual behavior is mixed (Abula et al., 2016), knowledge of official (e.g., national) recommendations for PA could make it easier for individuals to assess the extent to which they engage in PA (Knox et al., 2015) and might be a prerequisite for behavior change. In a study exploring PA for preventative health in dancers (Hanna et al., 2017), respondents scored low on a questionnaire assessing their knowledge of public health messages in which they were asked to rate the extent to which they agreed with items such as “Taking the stairs at work or generally being more active for at least 30 min each day is enough to improve your health” and “Exercise doesn’t have to be done all at one time – blocks of 10 min are okay.”

### Barriers to Engagement in PA and Theoretical Framework

Many interventions involving PA produce results with moderate effect sizes. Few reports of such interventions include the theoretical framework underpinning the intervention, and even fewer discuss the stages through which the design of a theory-based intervention developed (Taylor et al., 2013). Existing theories of behavior, such as the Health Belief Model, the Theory of Reasoned Action, the Theory of Planned behavior, and the Social Cognitive Theory are often used to understand behavior, rather than behavior change.

Barriers to and enablers of any behavioral change must be determined if an intervention is to be tailored to the needs of a specific group at multiple levels (e.g., the individual’s physical and psychological capability for change, the environment in which they live and work, and how motivated they are to change) and, therefore, more likely to be effective (National Institute for Health and Care Excellence, 2014). For example, findings from focus group interviews suggest that university students’ levels of PA are affected by a complex interaction of individual factors such as enjoyment and time, with the physical environment in terms of accessibility, travel time and prices, and the macro environment represented by the media and advertising (Deliens et al., 2015).

Our study used the Theoretical Domains Framework (TDF) to explore the determinants of engagement in PA (Michie et al., 2008). The TDF is an integrative model that brings together other existing theoretical approaches and, on the basis of expert consensus, distils overlapping concepts into a set of 11 determinants of behavior change, including concepts such as the environment, emotion, motivation, beliefs about capabilities, and social influences, which function as a guide to exploring what might affect any behavior. These determinants are assessed to indicate potential areas of strength and weakness. Based on this evaluation, specific behavior change techniques (BCTs) can then be matched to various determinants, enabling interventions to be tailored to groups or individuals as required.

### Sedentary Behavior

Sedentary behavior has been defined as any waking activity requiring an energy expenditure lower than or equal to 1.5 metabolic equivalents (METs) while sitting or lying down (Sedentary Behaviour and Research Network, 2012). Sitting for too long is not just a symptom of insufficient PA, but has also been associated with multiple health issues. Whether musicians are sitting or standing while practicing and performing could affect physiological stress and health risks (Spahn et al., 2014). Although prolonged sitting has been associated with musculoskeletal pain in office workers (Gupta et al., 2015; Hallman et al., 2015), the evidence remains limited and inconclusive, as PA is not always taken into consideration as a confounder (de Rezende et al., 2014; van der Ploeg and Hillsdon, 2017). Playing a musical instrument such as the violin while sitting is characterized by approximately 2.0 METs (Manchester, 2011), so although this does not adhere to the definition of sedentary behavior given above, little is known about musicians’ sitting and PA when they are at leisure. Recent research conducted among elite athletes including footballers and rowers suggests that, while they exceed the recommended levels of PA generally, they are often sedentary during their leisure time (Judice et al., 2014; Weiler et al., 2015; Sperlich et al., 2017). The implications of this pattern of behavior remain unknown.

### The Present Study

Many studies investigating risk factors for musculoskeletal problems among musicians have methodological limitations and their findings cannot therefore support the identification of causal relationships (Wu, 2007; Baadjou et al., 2016; Rotter et al., 2019), particularly as musculoskeletal problems are likely to have multiple causes that may interact with each other. However, the process of identifying potential causal interactions must begin by finding associations between them (Woldendorp et al., 2018). While the study reported here was also cross-sectional and correlational only, it aimed to investigate, independently and in relation to each other, a set of potential risk factors for PRMDs identified in the literature to date. These include practice time and practice-related preventative behaviors such as warming up, on and away from the instrument, and taking breaks; engaging in PA; and anxiety. Additionally, it aimed to explore, for the first time, the relationship between sedentary behavior and PRMDs. Determinants of and barriers to engagement in PA were investigated independently to understand better what might prevent music students from engaging in PA. Self-reported pain and perceived exertion were investigated because, like physical strain and muscle fatigue, they are associated with PRMDs (Ackermann et al., 2012). Additionally, the study explored the information about risk factors for PRMDs that students had received as part of their training, and the sources of that information.

No hypotheses were generated as the study was exploratory. Instead, the following research questions were asked:

• What is the students’ knowledge of official guidelines for PA?
• What are students’ self-reported levels of PA including muscle-strengthening exercise?
• What are the barriers to and determinants of engaging in PA?
• How much sedentary behavior do they report, both occupational (when playing their instrument) and non-occupational?
• To what extent have they learned about risk factors for PRMDs and the importance of PA during their training?
• From what sources have they found out about risk factors for PRMDs?
• How do they believe PRMDs can be prevented and what strategies have they found effective themselves?
• What is their experience of anxiety, in terms of its intensity?
• What relationships are there between students’ experience of pain, PRMDs, anxiety, practice behaviors, PA, and sedentary behavior?

## Materials and Methods

### Design

The design of the study was a cross-sectional questionnaire survey.

### Respondents

Respondents were undergraduate and postgraduate students (PGs) at UK conservatoires. They were recruited using opportunity sampling, via various routes: at orchestral rehearsals, and at sessions organized particularly for the purpose of recruiting and administering the questionnaire; via social media and emails sent to conservatoire administrators; and via the first author’s personal contacts.

### Questionnaire

The questionnaire that was administered incorporated items to collect demographic data, standardized questionnaires, items excerpted or adapted from standardized questionnaires, and items created by the first author (see Supplementary DataSheet S1). The standardized questionnaires were the International Physical Activity Questionnaire – Short Form (IPAQ-SF); Barriers to Being Physically Active quiz (Centers for Disease Control [CDC], 1999); Determinants of Physical Activity Questionnaire (DPAQ: Taylor et al., 2013); Sedentary Behavior Questionnaire (SBQ: Rosenberg et al., 2010); Rating of Perceived Exertion Scale (Borg, 1998); and the anxiety scale of the Hospital Anxiety and Depression Scale (HADS: Zigmond and Snaith, 1983). Two items on pain were excerpted from the RAND 36-Item Short Form Survey Instrument (SF-36: Ware and Sherbourne, 1992; McDowell, 2006). Two items on PRMDs were adapted from Ackermann and Driscoll (2010), and two items on warming up, two on taking breaks, and five on training advice were adapted from Davies and Mangion (2002). Four items on knowledge of PA guidelines were adapted from Knox et al. (2015). Finally, two items on health-related training in relation to PA were created by the researcher for the purpose of this study.

#### Demographic Data

It consisted of sex, age, nationality, degree, name of conservatoire, nature of genre studied (classical or popular music), and academic level (undergraduate or postgraduate).

#### Practice and Warming Up

Items included the name of main instrument; the number of years playing main instrument; number of hours spent in a typical week in individual practice; the frequency and length of taking breaks during practice sessions; the duration of a practice session before taking a break; and the frequency of warming up on the instrument (via slow scales, long tones, and finger exercises) and away from it (via movement, stretching, cardiovascular, or core muscle movement) before practicing or playing, adapted from Davies and Mangion (2002).

#### Health-Related Training

A yes/no item was included on whether participants had received any information on how to prevent health-related pain and, if so, the source(s) of the information. Then they were asked whether or not they found it easy to access such information, and if they had received advice during their training on nine items related to the prevention of PRMDs, of which five were adapted from Davies and Mangion (2002). These included warming up on and away from the instrument, and taking breaks during playing. The first author added two items on engaging in aerobic PA and muscle strengthening exercises.

#### Knowledge of PA Guidelines

Recognition is easier than recall from memory so, rather than providing multiple-choice options, open-ended questions adapted from Knox et al. (2015) about officially recommended amounts of aerobic PA and muscle strengthening were included. Respondents who answered “yes” to the question “Do you know what the national recommendations are for taking part in PA, in terms of minutes per week of moderate intensity PA?” were prompted to state the national recommendations in terms of minutes per week. The correct answer is 150 min per week; respondents who estimated fewer and more than 150 min were labeled over- and under-estimators, respectively. The same format was used to evaluate knowledge of guidelines regarding muscle strengthening exercises in days per week (the correct answer is “two”).

#### Reported Physical Activity

The IPAQ-SF was used since it has reasonable measurement characteristics for PA (Craig et al., 2003). Respondents were asked how much time they had spent walking, and doing moderate and vigorous PA over the previous 7 days. Response options were numbers of hours and/or minutes, subsequently computed in minutes only. All cases in which variables representing walking, moderate, and vigorous PA exceeded 180 min (3 h) were recoded as 180 min, while variables representing walking, moderate, and vigorous PA exceeding 1260 min (21 h) were recoded as 1260 min so that a realistic maximum of 21 h (3 h ^∗^ 7 days) of PA was allowed for each respondent (IPAQ, n.d.). Metabolic equivalent of energy expenditure (MET)-minutes/week values were computed for each type of activity according to the following pattern: 3.3 for walking, 4.0 for moderate, and 8.0 for vigorous PA. Next, MET-minutes/week scores were calculated (e.g., Walking MET-minutes/week = 3.3 ^∗^ walking minutes ^∗^ walking days). The total sum of MET-minutes/week was thus computed by adding the values of MET-minutes/week for walking, moderate, and vigorous activity. Cut-off scores were also used, so respondents could be categorized as engaging in low, moderate, and high PA defined as follows:

• Low – Individuals not meeting the criteria for “moderate” or “high.”
• Moderate – Individuals who satisfied one of the following conditions: (a) three or more days of vigorous-intensity activity of at least 20 min per day OR (b) five or more days of moderate-intensity activity and/or walking of at least 30 min per day OR (c) five or more days of any combination of walking, moderate-intensity, or vigorous intensity activities achieving a minimum total PA of at least 600 MET-minutes/week.
• High – Individuals who satisfied one of the following conditions: (a) vigorous-intensity activity on at least 3 days achieving a minimum total PA of at least 1500 MET-minutes/week OR (b) seven or more days of any combination of walking, moderate-intensity, or vigorous-intensity activities achieving a minimum total PA of at least 3000 MET-minutes/week.

One further item was added by the first author to find out how many respondents had engaged in specific exercises for muscle strengthening exercises using their own body (e.g., yoga, sit-ups, or push-ups), weights, or resistance bands during the previous month.

#### Barriers to Engaging in Physical Activity

The Barriers to Being Physically Active quiz (Centers for Disease Control [CDC], 1999) is a 21-item survey encompassing (1) lack of time, (2) social influence, (3) lack of energy, (4) lack of willpower, (5) fear of injury, (6) lack of skill, and (7) lack of resources. Response options range from 0 – very unlikely to 3 – very likely. According to the instructions, a score of 5 or more for any of these categories indicates a considerable barrier. The DPAQ derives from the TDF (Taylor et al., 2013) and contains 34 items matched to 11 factors: (1) knowledge, (2) environmental context and resources, (3) motivation and goals, (4) beliefs about capabilities, (5) skills, (6) emotion, (7) social influences, (8) beliefs about consequences, (9) action planning, (10) coping planning, and (11) goal conflict. The questionnaire has good discriminant validity and test–retest reliability, and reasonable internal consistency for most factors. Eight of these determinants differentiate reliably between high and low exercisers.

#### Sedentary Behavior

The SBQ asks participants how much time they spent “from when you wake up until you go to bed” engaging in each of nine sedentary behaviors during a typical weekday and a typical weekend day: watching TV, playing computer or video games, sitting listening to music, sitting and talking on the phone, doing paperwork or computer work, sitting reading a book or magazine, playing a musical instrument, doing artwork or crafts, and sitting and driving in a car, bus, or train. Response options were none, 15 min or less, 30 min, 1 h, 2 h, 3 h, 4 h, 5 h, and 6 h or more. Each response was converted into hours (e.g., 30 min was recoded as 0.5 h). Total numbers of hours of sedentary behavior were summed separately for weekday and weekend days for each item. Next, weekly scores were computed by multiplying weekday hours by 5 and weekend hours by 2 and summing the two results. Responses representing more than 24 h/day were recoded as 24 h/day for variables of total hours/day (weekday and weekend) and total hours/week. Also, variables were created to sum the number of hours spent every day in all listed behaviors for weekday and weekend days separately. Next, weekly estimates were calculated by multiplying weekday hours by five and weekend day hours by two. Finally, another variable was created for the total number of hours spent in sitting behaviors per week. Answers representing more than 24 h/day were coded as 24 h/day (Rosenberg et al., 2010).

The SBQ has acceptable measurement properties for adults (Rosenberg et al., 2010). Both weekday and weekend day TV viewing showed excellent reliability (ICC = 0.86, 95% CI [0.76–0.92]) and ICC = 0.83, 95% CI [0.72–0.90], respectively: Prince et al., 2017). Mean, median, and standard deviation values are reported for the following: hours/week for each activity; total sedentary hours/week; total weekday (hours/day); total weekend (hours/day) (Rosenberg et al., 2010). In order to look at non-occupational scores, “playing a musical instrument” was removed.

Pain was measured via two SF-36 items on intensity (from 1 – none to 6 – very severe) and the extent to which it had interfered with the respondent’s practice and performance “during the last 4 weeks” (from 1 – not at all to 5 – extremely). The latter item was adapted from the original “During the past 4 weeks, how much did pain interfere with your normal work (including both work outside the home and housework)?”

Performance-related musculoskeletal disorders were investigated in terms of frequency and severity through two items adapted from Ackermann and Driscoll (2010), measured on 11-point Likert scales, from 0 – never to 10 – constantly, and from 0 – none to 10 – most severe, respectively.

One item asked about perceived exertion, defined as the amount of physical effort respondents reported needing to complete their daily practice routines over the preceding seven days, provided these represented a typical week: the Rating of Perceived Exertion Scale (Borg, 1998), which ranges from 6 – no exertion at all to 20 – maximal exertion.

#### Anxiety

The seven-item anxiety scale from the HADS was used. An anxiety score was computed. Total scores were labeled as normal (0–7), borderline abnormal (8–10), or abnormal (11–21).

### Procedure

Ethical approval was granted by the Conservatoires UK Research Ethics Committee. The questionnaire was administered both online and via hard copies between June 2017 and April 2018 to music students at all UK conservatoires, although no responses were received from the Royal Birmingham Conservatoire or Leeds College of Music.

### Data Treatment and Analyses

This study followed a cross-sectional and correlational design. Data from music students were analyzed by sex and different levels of study using IBM SPSS v.22. The internal reliability of some of the scales was quantified using Cronbach’s *alpha*.

According to the visual analysis of histograms, and Kolmogorov–Smirnoff tests, most of the variables were not normally distributed, so non-parametric statistical tests were applied. Analyses examining differences between groups were carried out using the Mann–Whitney *U*-test. Associations between variables of interest were explored through the non-parametric Spearman’s *rho*, bootstrapped (bias-corrected and accelerated BCa) 95% confidence intervals (Field, 2013) were calculated, and the alpha level was set at 5%.

## Results

### Descriptive and Inferential Statistics

Demographic information was obtained from 111 students, aged 18–31, median (MD) = 22, of whom 64 (58%) were female. Ninety-three respondents (84%) were undergraduate students (UGs). Eighty-two respondents (74%) were from the Royal Northern College of Music, 18 (16%) were music students from Trinity Laban Conservatoire of Music and Dance, and the remainder were from the Guildhall School of Music and Drama, Royal Welsh College of Music and Drama, the Royal College of Music, Royal Academy of Music, and Royal Conservatoire of Scotland. Seventy-three (68%) students were from the United Kingdom, 22 (20%) from Europe, seven (6.5%) were from Asia, four (4%) were from Australia or New Zealand, and two (2%) were American. In terms of instrument/school of study, 51 (47%) were string players, 31 (28%) were wind and brass players, 13 (12%) were singers, 10 (9%) were keyboard players, two (2%) were percussionists, and two (2%) were composers.

Fifty-nine respondents completed the section on practice and warming up. They reported playing their instruments for a mean of 11.74 h in a typical week (*SD* = 4.04; range: 3–24) (statement to be removed). The total number of hours spent in individual practice was a mean of 19.50 (*SD* = 8.26; range: 3–42). Breaks lasted a mean of 12 min (*SD* = 11.47; range: 2–60) and respondents reported practicing for a mean of 44 min before taking a break (*SD* = 20; range: 10–90). The questionnaire asked about frequency of warming up before practicing and playing, on the instrument (through slow scales, long tones, and finger exercises), and away from it (through movement, stretching exercises, cardiovascular or core muscle movement). As shown in Table 1, out of 111 respondents, 96 (86.5%) reported warming up on the instrument “quite frequently” and “very frequently” while only 44 (39.6%) reported doing so away from the instrument.

**TABLE 1:** Frequency of warming up on and away from the instrument.

***N* = 111**	**Never**	**Occasionally**	**Quite frequently**	**Very frequently**
Warming up ON your instrument	4 (3.6%)	11 (9.9%)	30 (27%)	66 (59.5%)
Warming up AWAY from your instrument	25 (22.5%)	42 (37.8%)	25 (22.5%)	19 (17.1%)
				

One hundred and ten respondents completed the section of the questionnaire asking if they had received specific health-related advice during their training. Table 2 shows the number and percentage who responded “yes” to each item.

**TABLE 2:** Health-related advice received during training.

***N* = 110**	***n* who said yes (%)**
How to warm up (on your instrument)	103 (94%)
How to play with flexibility and free movement	95 (86%)
How to sit comfortably when playing (including correct chair height, type of chair, position of stand)	84 (77%)
When to take breaks during playing and practice sessions	87 (79%)
How to look after your muscles and prevent strain	79 (72%)
How to pace yourself during periods of intensive practice and playing	77 (70%)
How to warm up (away from your instrument)	73 (66%)
Why you should engage in aerobic/cardio physical activity	66 (62%)
Why you should do muscle strengthening exercises	60 (57%)

As shown in Table 3, 88 (80% of 110) respondents had been given information on how to prevent playing-related pain. The source of information was usually the respondent’s teacher (43%), although 25% mentioned a lecture or the college, and 23% mentioned a health professional. Ten (9%) mentioned books, magazines, and websites or the British Association of Performing Arts Medicine (BAPAM). Out of 108 respondents, 64 (60%) said they found it easy to access information about preventing/treating PRMDs, while 44 (40%) did not.

**TABLE 3:** Sources of information on pain prevention.

**Source (*N* = 110)**	***n* of participants who mentioned it (%)**
Teacher	48 (43%)
Lecture/college	28 (25%)
Health professional (physiotherapist, doctor, GP, osteopath, chiropractor, Alexander Technique instructor, Pilates instructor)	26 (23%)
Books, magazines, websites	7 (6%)
BAPAM	3 (3%)

More than three-quarters of respondents (83 or 76.1% of 109 respondents) said they did not know the national recommendations for taking part in PA. Of the 26 (23.9%) respondents who said they did know them, eight (31%) gave correct responses, 10 were over-estimators (38.5%), and eight (31%) were under-estimators. The vast majority (103 or 94.5%) did not know the national recommendations for muscle strengthening exercises. Of the six who did, three gave the correct answer while the other three were over-estimators. Fifty-seven respondents, two of whom preferred not to disclose their sex, completed the IPAQ-SF measuring engagement in PA. Cronbach’s *alpha* of 0.75 was found for all three scales and can be regarded as acceptable. As shown in Table 4, 40 (70%) respondents reported moderate PA, while 15 (26%) reported high PA. When divided by sex, no male and two (5.6%) females reported low PA; 14 (74%) males and 25 (69%) females reported moderate PA; and five (26%) males and nine (25%) females reported high PA. As for program, two (5%) UGs and no PGs reported low PA; 26 (65%) UGs and 14 (82%) reported moderate PA; and 12 (30%) UGs and three (18%) PGs reported high PA.

**TABLE 4:** Physical activity.

	***N* = 57 (%)**	**Males (*n* = 19)**	**Females (*n* = 36)**	**UG (*n* = 40)**	**PG (*n* = 17)**
Low	2 (3.5%)	0	2 (5.6%)	2 (5.0%)	0
Moderate	40 (70.2%)	14 (73.7%)	25 (69.4%)	26 (65.0%)	14 (82.4%)
High	15 (26.3%)	5 (26.3%)	9 (25%)	12 (30.0%)	3 (17.6%)

As shown in Table 5, mean total PA per week was 2326 MET-minutes (*SD* = 1846), of which 606 MET-minutes (*SD* = 1107) were contributed by vigorous activity, 345 MET-minutes (*SD* = 570) by moderate activity, and 1375 (*SD* = 1012) MET-minutes by walking. Median minutes/week and MET-minutes/week, and inter-quartile ranges, were calculated because the data were not normally distributed and there were outliers. Mann–Whitney *U*-tests were conducted to compare the walking, moderate, vigorous, and total PA of men vs. women and UG vs. PGs, but there were no significant differences between groups.

**TABLE 5:** Types of physical activity.

		**Mean (*SD*) (*N* = 57)**	MD (IQR)* (*N* = 57)
Vigorous	Mins/week	75 (138)	5 (0–90)
	MET-min/week	606 (1107)	40 (0–720)
Moderate	Mins/week	86 (142)	30 (0–120)
	MET-min/week	345 (570)	120 (0–480)
Walking	Mins/week	416 (306)	420 (180–450)
	MET-min/week	1375 (1012)	1386 (594–1485)
Total PA	Mins/week	578 (398)	440 (330–840)
	MET-min/week	2326 (1846)	1674 (1386–2874)

*Median (interquartile range).

Twenty-nine respondents completed the item on muscle strengthening exercises, reporting a median and mean of three times per week (range: 0–6.50; *SD* = 1.78). A total of 106 respondents completed the items representing barriers to being active. As shown in Table 6, the following barriers received mean scores of more than 5: lack of time, energy, willpower and resources; and social influence.

**TABLE 6:** Barriers to being active.

**Barriers (*N* = 106)**	**Mean score (*SD*)**
Lack of time*	8.44 (2.50)
Lack of energy*	8.06 (2.55)
Lack of willpower*	7.61 (2.91)
Social influence*	6.23 (2.31)
Lack of resources*	5.96 (2.36)
Lack of skill	4.77 (2.25)
Fear of injury	4.10 (1.82)

*Barriers that received a score higher than 5.

Fifty-two respondents completed the items representing determinants of PA. As shown in Table 7, the majority received mean scores around the mid-point of 3.5 (range: 3.34–6.11) but those items receiving mean scores above 5 (Emotion, Action Planning, Environmental Resources and Beliefs about Consequences) were less likely to be barriers.

**TABLE 7:** Determinants of physical activity.

**Determinants (*N* = 52)**	**Mean score (*SD*)**
Beliefs about consequences	6.11 (0.93)
Environmental resources	5.67 (1.20)
Action planning	5.07 (1.57)
Emotion	5.05 (1.66)
Skills	5.00 (1.53)
Social influences	4.83 (1.36)
Motivation goals	4.81 (1.39)
Beliefs about capabilities	4.11 (0.85)
Knowledge	3.81 (1.50)
Coping planning	3.44 (1.65)
Goal conflict	3.34 (1.48)

Data on sedentary behavior were provided by 105 respondents. As shown in Table 8, respondents reported being sedentary, other than when they were playing their instruments, for a mean of 5.51 (*SD* = 3.25) hours per day during the week and 6.52 (*SD* = 2.95) hours per day at the weekend. Medians and inter-quartile ranges were calculated, because the data were not normally distributed and outliers were present.

**TABLE 8:** Sedentary behavior.

***N* = 105**	**M (*SD*)**	**MD (IQR)**		
TV	9.56 (7.64)	9.00(4−14)	“Play musical instrument” removed – M (SD)	“Play musical instrument” removed – MD (IQR)
Computer games	1.88 (4.59)	0(0−1.75)		
Listen to music	7.81 (8.02)	4.50(2.25−11)		
Talk on phone	3.13 (3.00)	2.25(1.75−3.50)		
Paper work	8.27 (7.43)	6.00(3−12)		
Reading	4.13 (4.49)	3.00(1−6)		
Play musical instrument	23.48 (9.81)	24.00(19−30)		
Arts and crafts	1.45 (3.24)	0(0−1.75)		
Driving in a car/bus/train	4.34 (5.44)	2.25(0−7)		
Total SB h/w*	64.10 (24.79)	62.25(48−74.25)	40.61 (20.98)	37.75(26.50−47.75)
Total SB weekday h/day	9.00 (3.83)	8.50(7−10)	5.51 (3.25)	5.00(3.50−6.50)
Total SB weekend h/day	9.55 (3.50)	9.25(7−11.50)	6.52 (2.95)	6.00(4.50−8.50)

*SB h/w = sedentary behavior hours/week

Postgraduate students spent significantly more time (*M* = 7.5 [*SD* = 7.08] hours per week) than UGs sitting while driving (*M* = 3.73, *SD* = 4.89; *U* = 574.500, *p* = 0.04), while UGs spent significantly more time (*M* = 3.46, *SD* = 3.12) talking on the phone than PGs (*M* = 1.44, *SD* = 1.42; *U* = 521.500, *p* = 0.01). Fifty-nine respondents completed the section of the questionnaire on reported pain, which used scales of 1–6 for intensity of pain experienced in the previous month and 1–5 for the extent to which it interfered with their practice and performance. Mean scores were 2.81 (*SD* = 1.15) and 1.97 (*SD* = 0.94), respectively. One hundred and eight respondents reported a mean frequency of PRMDs of 3.98 (*SD* = 2.72) while 109 respondents reported a mean severity of 3.72 (*SD* = 2.35), both measured on scales of 0–10. Both variables were also transformed into dichotomous variables such that frequency and severity were labeled “low” if they were rated 5 or less, and “high” if they were rated 6 or more. Eighty-five respondents (79%) reported low frequency and 96 (88%) reported low severity of PRMDs. One hundred and ten respondents completed the Borg Reported Perceived Exertion scale. The mean score was 7.60 (*SD* = 2.57) corresponding to between 12 and 13 (“somewhat hard”on the RPE scale). Fifty-six respondents completed the Anxiety component of the HADS. The scale showed good internal reliability (Cronbach’s *alpha* = 0.82). The mean score was 9.57 (*SD* = 4.90), classed as borderline abnormal. When using categories according to the suggested cut-off scores, 34% of respondents were found to report normal (mean 0–7), 23% borderline abnormal (mean 8–10), and 43% abnormal levels of anxiety (mean 11–21).

### Relationships Between Variables of Interest

Relationships were explored between six sets of variables:

1. Pain: general and interfering with practice and performance
2. PRMDs: frequency and severity
3. Anxiety: general (measured on the HADS scale)
4. Practice behaviors: time (hours per week), frequency of breaks, length of session before taking a break, warming up on, and away from the instrument
5. PA: reported physical exertion (RPE), total PA (minutes per week), muscle-strengthening exercises
6. (Non-occupational) sedentary behavior: weekday, weekend, total.

Correlations between all variables using Spearman’s *rho* were calculated and are shown in Table 9. Cases were excluded pairwise to deal with missing values.

**TABLE 9:** Correlations between variables.

	**1**	**2**	**3**	**4**	**5**	**6**	**7**	**8**	**9**	**10**	**11**	**12**	**13**	**14**	**15**
1. Bodily pain (*n* = 59)	–														
2. Bodily pain interfering (*n* = 59)	0.686**	–													
3. PRMDs frequency (*n* = 108)	0.603**	0.733**	–												
4. PRMDs severity (*n* = 109)	0.562**	0.707**	0.885**	–											
5. RPE (*n* = 110)	0.205	0.345**	0.279**	0.186	–										
6. Practice time (hours/week) (*n* = 58)	0.176	0.236	0.128	0.097	0.113	–									
7. Taking breaks frequency (*n* = 59)	0.082	0.090	–0.030	–0.071	0.127	–0.030	–								
8. Length of practice session before breaking (*n* = 59)	–0.067	0.084	0.038	–0.110	0.142	0.399**	−0.294*	–							
9. Warming up ON instrument frequency (*n* = 111)	0.169	–0.083	–0.143	–0.185	–0.028	0.050	–0.167	0.155	–						
10. Warming up AWAY from instrument frequency (*n* = 111)	0.170	0.084	–0.134	–0.047	–0.071	0.084	0.278*	–0.083	0.110	–					
11. Total PA mins/week (*n* = 57)	–0.034	–0.136	–0.127	–0.015	–0.082	–0.259	0.114	–0.201	0.018	0.294*	–				
12. Muscle strengthening (times/week) (*n* = 29)	0.088	0.135	–0.131	0.099	0.072	0.355	–0.033	0.159	0.149	0.304	0.241	–			
13. Anxiety (HADS) (*n* = 56)	0.343**	0.341*	0.438**	0.340*	0.335*	–0.018	0.005	0.008	–0.078	0.198	–0.020	0.106	–		
14. Weekday non-occupational SB (*n* = 107)	–0.116	–0.138	0.135	0.176	–0.054	−0.387**	–0.170	−0.305*	–0.166	0.024	–0.092	−0.539**	0.150	–	
15. Weekend non- occupational SB (*n* = 107)	0.035	0.056	0.213*	0.217*	0.036	–0.174	–0.162	–0.162	–0.177	–0.044	–0.123	−0.489**	0.199	0.768**	–
16. Total non- occupational SB (*n* = 109)	–0.073	–0.099	0.162	0.188	–0.047	−0.348**	–0.159	−0.280*	–0.176	–0.024	–0.102	−0.581**	0.161	0.970**	0.876**

*p < 0.05 and **p < 0.01.

First, significant associations between variables within each set, with bootstrapped (bias-corrected and accelerated BCa) 95% confidence intervals (Field, 2013) are reported. There were associations between bodily pain and bodily pain interfering with practice and performance (*r_*s*_* = 0.686, CI = 0.494–0.823, *p <* 0.001) and the frequency and severity of PRMDs (*r_*s*_* = 0.885, CI = 0.803–0.938, *p <* 0.001). Hours of practice per week were positively associated with length of practice session before taking a break (*r_*s*_* = 0.399, CI = 0.161–0.607, *p <* 0.001), and there were positive associations between total, weekday (*r_*s*_* = 0.970, CI = 0.947–0.982, *p <* 0.001), weekend (*r_*s*_* = 0.768, CI = 0.657–0.850, *p <* 0.001), and weekday and weekend hours of non-occupational sedentary behavior (*r_*s*_* = 0.876, CI = 0.812–0.918, *p <* 0.001).

Second, significant associations between sets of variables were as follows: Bodily pain interfering with practice and performance was associated with frequency (*r_*s*_* = 0.733, CI = 0.579–0.849, *p <* 0.001) and severity of PRMDs (*r*_*s*_ = 0.707, CI = 0.538–0.826, *p <* 0.001), anxiety (HADS: *r*_*s*_ = 0.438, CI = 0.089–0.562, *p <* 0.001), and RPE (*r*_*s*_ = 0.345, CI = 0.092–0.464, *p <* 0.001). Frequency of PRMDs was associated with anxiety (HADS: *r_*s*_* = 0.438, CI = 0.168–0.661, *p <* 0.001), RPE (*r_*s*_* = 0.279, CI = 0.091–0.464, *p <* 0.001) and sedentary behavior at the weekend (*r_*s*_* = 0.213, CI = 0.007–0.405, *p <* 0.05). Severity of PRMDs was also associated with anxiety (HADS: *r_*s*_* = 0.340, CI = 0.078–0.572, *p <* 0.001) and sedentary behavior at the weekend (*r_*s*_* = 0.217, CI = 0.033–0.379, *p <* 0.05) but not RPE. This in turn was associated with anxiety (HADS: *r_*s*_* = 0.335, CI = 0.107–0.543, *p <* 0.05). Hours of practice per week were negatively associated with weekday (*r_*s*_ = −*0.387, CI = *−*0.647 to *−*0.094, *p <* 0.001) and total non-occupational sedentary behavior (*r_*s*_ = −*0.348, CI = *−*0.602 to −0.055, *p <* 0.001). Frequency of taking breaks was negatively associated with length of practice session before taking a break (*r_*s*_* = −0.294, CI = −0.505 to −0.052, *p* < 0.05) and positively associated with warming up away from the instrument (*r_*s*_* = 0.278, CI = 0.044–0.503, *p <* 0.05). Length of practice session before taking a break was also negatively associated with weekday (*r_*s*_* = −0.305, CI = −0.534 to −0.042, *p <* 0.05) and total non-occupational sedentary behavior (*r_*s*_* = −0.280, CI = −0.536 to −0.019, *p <* 0.05). Warming up away from the instrument was associated with total PA [minutes per week: (*r_*s*_* = 0.294, CI = 0.066–0.491, *p* < 0.05)]. Engaging in muscle-strengthening exercise was negatively associated with total non-occupational sedentary behavior (*r_*s*_* = −0.581, CI = −0.783 to −0.289, *p <* 0.001), on weekdays only (*r_*s*_* = −0.539, CI = −0.746 to −0.253, *p <* 0.001) and at weekends only (*r_*s*_* = −0.489, CI = −0.741 to −0.162, *p <* 0.001).

## Discussion

In this study, we investigated variables that have been associated with risk factors for PRMDs, including practice-related strategies such as taking breaks and warming up, self-reported levels of PA and anxiety. We explored the interactions between them and with PRMDs, pain and perceived exertion. We also explored respondents’ knowledge of PA guidelines, barriers to PA engagement, and whether respondents might be receiving relevant health-related information and from what sources. Finally, we looked at levels of various forms of sedentary behavior and its interaction with other variables.

### Descriptive Data

Respondents tend to practice for about 45 min before taking a break that lasts a few minutes on average. This is in line with current recommendations, although some authors recommend a 5-min rest break every 25 min of playing (e.g., Chan and Ackermann, 2014). While most music students report engaging in warming up on the instrument frequently, considerably fewer do so away from the instrument. This is supported by other research findings. While up to 72% musicians report engaging in musical warm-ups, only 18% carry out physical warm-ups (Zaza and Farewell, 1997). The evidence on the benefits of doing so remains inconclusive and under-investigated (McCrary et al., 2015). Only one study conducted with 55 violinists compared the effects of various forms of warming up both on the instrument and away from it (cardiovascular and core muscle warming-up) and found they were equally effective in lowering perceived exertion when compared to an inactive control group (McCrary et al., 2016). However, no particular warm-up activity had any objectively measurable effect on muscle activity levels. It is thus unclear whether there are important distinctions between different types of warm-up activities. Of course, while all the practice-related strategies discussed above might describe the respondents’ typical week, these patterns can fluctuate during stressful times such as audition preparation, considerable playing demands, and various other types of performance (Chan and Ackermann, 2014).

Respondents who had received health-related advice during training were most likely to have received information on warming up on the instrument (94%) but considerably less likely to have received information about warming up away from the instrument (66%). Even fewer had been told why they should engage in aerobic/cardiovascular PA (62%) and muscle-strengthening exercises (57%). Although PRMDs are complex phenomena, subject to multiple influences, and there is not much literature on the topic, engaging in PA could be an effective strategy for lowering the risk of PRMDs. However, as mentioned earlier, the evidence is mixed.

When asked to state sources of health-related information, most respondents mentioned their teachers and lectures at college. As 77% of 192 piano students mentioned music educators as the main source of their awareness of PRMDs (Ling et al., 2016), this once again highlights the important role of higher music education institutions, and teachers in particular, in health education. Only 9% of respondents in the present study mentioned websites or BAPAM as a source of information. It may be that in addition to music students’ not receiving much training on certain relevant issues such as the importance of PA, more effort should be invested in making such information relevant and/or attractive so as to engage them. After all, 40% of respondents reported not finding it easy to access information about PRMDs.

Respondents’ knowledge of PA guidelines for healthy adults was especially poor, even though most met and in some cases exceeded the recommended 150 min per week. This seems to replicate a similar pattern found among university dance students (Hanna et al., 2017). More than a third of those who thought they knew the official PA guidelines over-estimated the actual figure. On one hand, over-estimating the guidelines – for example, thinking that PA means only *going* to the gym, rather than actually engaging in PA while there – could be a barrier. On the other hand, engagement in only 150 min per week of PA, while certainly to be encouraged in the general population for a range of health benefits, may be insufficient for preventing PRMDs in musicians. Given that most respondents completed the questionnaire online, they could have looked up the national recommendations for PA. Given that there were so few correct answers, they did not appear to have done so.

Respondents in this study reported comparable, albeit slightly lower, PA levels (except walking) to the levels reported by respondents in Araújo et al. (2020)’s study. Of those respondents, 79% exceeded the recommended weekly limits of PA, 10% met the recommendations, and 11% did not. In our study, only 26% exceeded recommended PA, 70% of respondents met the recommendations, and 3.5% did not. The distribution of mean total PA per week between vigorous, moderate PA, and walking was comparable to mean data from adults in 28 European countries, although musicians reported less vigorous and moderate exercise and considerably more walking (Gerovasili et al., 2015).

The percentage of people meeting the national guidelines for PA in the United Kingdom ranges from 19 to 76% (Loyen et al., 2016) while the prevalence of physical inactivity varies between 2 and 71% in more than 50 countries (Guthold et al., 2008) and between 23 and 44% in European countries (Sjöström et al., 2006; Gerovasili et al., 2015). These variations can be at least partially attributed to the use of self-report and different methods of assessment producing different results (Van Hecke et al., 2016). The respondents in Loyen et al.’s study reported a mean of 2543 MET-minutes/week, slightly more than than the mean of 2326 in the present study.

It is difficult to compare the results of the present study with those of previous studies because, even though a standard questionnaire was used, most questionnaires only have acceptable to moderate reliability and validity. Also, findings can be reported in different ways (e.g., total PA minutes/week, MET-minutes/week or in terms of categories: low, medium, and high; and although the IPAQ protocol recommends the use of median scores, means are often reported instead). Kapteyn et al. (2018) observe that respondents disagree on what constitutes PA and struggle to distinguish between moderate and vigorous intensities. Respondents might also consider the same activities as constituting both moderate and vigorous ones, thereby counting them twice. Nevertheless, it is cheaper and easier to administer questionnaires than to collect objective measures of PA.

The most notable barriers to engaging in PA reported by respondents in the present study were social influences, and lack of time, energy, willpower, and resources including access to facilities such as jogging trails, swimming pools and showers, and money. These barriers are similar to those identified by researchers internationally, including Arzu et al. (2006), Gómez-López et al. (2010), and Ashton et al. (2017). Social influences could be interpreted as insufficient endorsement of the benefits of PA at institutional, occupational, and social levels, while the other barriers reflect the busy lives led by music students. Environmental resources (facilities for PA, their geographical proximity, and the attractiveness of the local area), planning for and confidence while engaging in PA (“beliefs about consequences”) and goal conflict did not represent barriers. Taylor et al. (2013), who report similar mean scores, mean range, and hierarchy of barriers, hypothesize that goal conflict may not be an issue since multi-tasking is now so common.

Respondents in our study spent less time sitting, when not playing their instruments, than UK students who have been found, in other studies, to spend more than 8 h a day in sedentary behaviors including studying and watching TV (Nelson et al., 2008; Johnston et al., 2010; Rouse and Biddle, 2010; Deforche et al., 2015). Indeed, between 40 and 50% of university students worldwide are classed as physically inactive (Keating et al., 2005; Pengpid et al., 2015). Similarly, a comparison of musicians with athletes suggests that musicians are sedentary for less of their leisure time (Weiler et al., 2015).

Frequency and severity of reported PRMDs were similar to those reported in the existing literature, that is, rather low, while scores for reported perceived exertion were lower than those reported by Ackermann et al. (2002) and Chan et al. (2014a;b). Of course, it may be the case that students with few or no health issues were more likely than those currently experiencing them to complete the questionnaire. However, the present findings seem to also be in line with those of a multicenter study in which students at five German music universities were surveyed annually, for 3 years: more than 80% of respondents reported that experiencing few or no playing-related health problems over the course of their training (Spahn et al., 2017).

According to cut-off scores, 34% of respondents had “normal” levels of anxiety, 23% had “borderline abnormal,” and 43% had “abnormal” levels of anxiety. These levels were similar to those obtained from a sample of 69 musicians seeking or undergoing hand surgery in Germany (Spahn et al., 2001). Another study found that, in a sample of 239 music students in Germany, 33.5% had means above 8 on the anxiety scale, which are categorized as both borderline abnormal and abnormal. These scores were significantly higher than those obtained from medical and sports students (Spahn et al., 2004). This represents a proportion of respondents considerably lower than 66% whose levels of anxiety were categorized as borderline abnormal and abnormal in the present sample.

Overall, out of 110 respondents, 76 completed the questionnaires in hard copies and 34 online, via the Bristol Online Surveys. However, between March and April 2018, some RNCM students were asked to complete the set of questionnaires at the end of their lectures, in specifically designated slots. In order to match the length of these slots, the set of questionnaire was reduced, and so questions on engagement in PA, muscle strengthening, determinants of PA, anxiety, and pain were removed for those specific students, hence the smaller sample size for these items.

### Relationships Between Variables

The more hours respondents reported practicing each week, the longer they practiced in each session before taking a break and therefore the less often they took a break. Furthermore, the more often they took breaks, the more likely they were to warm up away from the instrument and engage in PA. Although causality cannot be inferred, it could be that students who practice more are less likely to use strategies for preventing PRMDs, such as taking breaks, and more likely to over-practice. It may also be that respondents considered breaks as a way of “warming up away from the instrument.” Further, the more respondents engaged in non-occupational sedentary behavior, the less they practiced and the shorter their practice sessions before taking a break. It is unclear what might explain these (admittedly weak) associations. Perhaps students who practice less and sit more are better at resting and relaxing, or perhaps practicing more motivates students to be more active when not practicing, although this contradicts the idea that practice can lead to more fatigue and thus less willingness and energy to engage in PA. Further investigation of these relationships is needed.

General anxiety measured on the HADS Scale was significantly and positively associated with frequency and severity of PRMDs, bodily pain, bodily pain interfering with practice and performance, and perceived exertion. Evidence for an association between anxiety and physical pain in musicians is inconsistent to date, so needs further investigation (Spahn et al., 2001; Davies and Mangion, 2002; Kaneko et al., 2005; Leaver et al., 2011; Kenny and Ackermann, 2015). It could be speculated that anxiety manifests itself via muscular tension experienced while playing thus jeopardizing performance and increasing the player’s anxiety (Davies and Mangion, 2002; Kaneko et al., 2005). Although causality cannot be inferred in the present study, anxiety could cause pain and *vice versa*. Additionally, there may be a third variable affecting both anxiety and pain.

The more respondents engaged in non-occupational sedentary behavior at weekends, the more frequent and severe were their reported PRMDs. Correlations were weak and the direction of the relationship is, of course, unclear. Perhaps more frequent and severe PRMDs forced respondents to be more sedentary when they could, or perhaps their symptoms resulted from insufficient muscle-strengthening PA.

No significant associations were found between practice-related strategies and PRMDs, pain or reported perceived exertion. Studies of relationships between playing time and musculoskeletal problems have produced mixed findings to date (e.g., Kaufman-Cohen and Ratzon, 2011; Kochem and Silva, 2017). Arguably the most reliable evidence suggests that warming up both on and away from the instrument is associated with reduced perceived exertion (McCrary et al., 2016). Other evidence to support popular recommendations is anecdotal and/or inconclusive (e.g., Yeung et al., 1999; Davies and Mangion, 2002). Although an association between engagement in PA and PRMDs has been both supported and disconfirmed (Yeung et al., 1999; Ling et al., 2018), no significant associations were found between PA and muscle strengthening and PRMDs, pain, perceived exertion, and/or anxiety. It may be that general PA is not be enough to support musical activities and/or target imbalanced muscles (Kenny and Ackermann, 2015). No optimal dose of PA has yet been agreed by health experts, including experts in musicians’ health.

Generally, non-significant results can be attributed to small sample sizes for certain variables, floor effects such as low scores for PRMDs, and ceiling effects such as high scores for PA; over-estimation was due, perhaps, to the recall effect and/or social desirability bias. Other limitations of this study include the lack of comparison data from objective measurements of PA (Adams et al., 2005; Chastin et al., 2014); use of non-standardized measures of PRMDs; and the length of the questionnaire, which could have led to response fatigue and, thus, lower reliability of respondents’ answers toward the end. The order of items was the same for all respondents so there could have been confounding effects of item order.

Studies using longitudinal designs are needed to establish causal relationships and disentangle temporal interactions between predictors and outcomes. This may enable complex interactions to be understood better and interventions to be designed that are more likely to be effective. The barriers to and determinants of PA most often identified in the present study could be investigated further via tailored questionnaires or explored qualitatively via interviews. Specific exercises rather than general levels of PA could be aimed at preventing PRMDs. It could also be useful to find out what motivates music students to engage in such activities (Taylor et al., 2013). Targeted interventions could be undertaken to improve health-related knowledge, particularly in relation to PA, as well as promoting evidence-based strategies for preventing and managing of PRMDs. Even though most respondents in the present study reported meeting or exceeding national recommendations for PA, this could have been the result of over-estimation. Furthermore, given the high levels of borderline abnormal and abormal anxiety in the present sample, strategies need to be put in place for preventing and managing anxiety.

Finally, Araújo et al. (2020) recommended that conservatoire training should be redesigned with “whole-system, context-driven, and comprehensive approaches” (p.15) (Healthy Conservatoires, n.d.). While this can mean many things and rigorous health education and promotion interventions should continue to be implemented, it is essential and high time that we also started questioning the ideology of Western classical music itself with its rigid set of norms (Leech-Wilkinson, 2020), so as to not just focus, in an arguably unethical manner, on making musicians stronger and more resilient for a professional world that is itself in need of serious change and never more so than now, since the beginning of the global pandemic that began at the end of 2019.

## Data Availability Statement

The raw data supporting the conclusions of this article will be made available by the authors, without undue reservation, to any qualified researcher.

## Ethics Statement

The studies involving human participants were reviewed and approved by the Conservatoires UK Research Ethics Committee. The patients/participants provided their written informed consent to participate in this study.

## Author Contributions

RM and JG designed the study and procedures and drafted the manuscript. RM collected and analyzed the data. Both authors edited the manuscript, read, and approved the submitted version.

## Conflict of Interest

The authors declare that the research was conducted in the absence of any commercial or financial relationships that could be construed as a potential conflict of interest.
