# Supplementary material for: Physical Activity, Sedentary Behavior, Anxiety, and Pain Among Musicians in the United Kingdom
Source: Front Psychol. 2020 Dec 3;11:560026. doi: 10.3389/fpsyg.2020.560026 (PMC7793824; doi:10.3389/fpsyg.2020.560026)
Supplement: Supplementary file 1 [file Data_Sheet_1.docx]

**Supplementary Data Sheet S1. The set of questionnaires. Physical activity, Sedentary Behaviour, Anxiety and Musculoskeletal issues Questionnaire**

As part of the Better Practice work package of the AHRC-funded CUK-wide research project Musical Impact ([www.musicalimpact.org](http://www.musicalimpact.org)), Raluca Matei (PhD candidate) and Professor Jane Ginsborg (RNCM) are investigating physical activity, anxiety and performance-related musculoskeletal disorders (PRMDs), and modifiable behaviours that might constitute risk factors including sedentary behaviour, and failure to warm up before practice sessions and take enough breaks when practising.

The survey is open to all students at any UK conservatoire, providing they play one or more instruments (i.e. not composers, conductors or singers), aged 18 years and above. Please complete this questionnaire and return it to the person who gave it to you by **15 December 2017**. It should take you about 25 minutes.

If you have any questions, please contact Raluca at [raluca.matei@student.rncm.ac.uk](mailto:raluca.matei@student.rncm.ac.uk), or Jane Ginsborg at [jane.ginsborg@rncm.ac.uk](mailto:jane.ginsborg@rncm.ac.uk).

By completing and submitting the questionnaire the researchers will assume that you have given your informed consent to take part in the research. You do not have to answer every question but it would be very helpful indeed for the research if you could do so.

Your responses to the questionnaire will remain anonymous. All the information that is collected about you will be kept strictly confidential. Any information about you that is disseminated will have your code identifier removed so you cannot be identified by it.

The results of the research will be reported in Raluca’s PhD thesis and related outputs such as conference proceedings and journal articles. They will also be used to design a behaviour change intervention and investigate the extent to which changes affect the risk of PRMDs.

Thank you for completing and submitting this questionnaire.

| **TODAY’S DATE :** |
| --- |

| Questions about you |
| --- |
| 2. Are you (please tick): Male Female Prefer not to say |
| 3. How old are you (years / months)? _________________________________ |
| 4. Nationality _________________________________ |
| 5. a) Degree:  ❑ BMus (Hons) Classical Music ❑ MMus (Hons) Classical Music ❑ Joint course students  ❑ BMus (Hons) Popular Music ❑ Other (please specify) .............................................  b) Where do you study?  ❑ Royal College of Music, London ❑ Guildhall School of Music and Drama  ❑ Trinity Laban Conservatoire of Music and Dance ❑ Royal Academy of Music  ❑ Birmingham Conservatoire ❑ Royal Northern College of Music  ❑ Royal Conservatoire of Scotland ❑ Royal Welsh College of Music and Drama  ❑ Leeds College of Music |
| 6. Current academic level:  ❑ Undergraduate ❑ Postgraduate |
| Your practice and preparation   1. What is your main instrument? ....................... 2. Total number of years playing main instrument............... (years) |
| Please answer the following questions with reference to the past 7 DAYS. (If the past 7 days do NOT represent a typical week, please think back to the most recent typical week.)  How much playing did you do (on all instruments) in the following categories?   1. Individual practice: ............. total hrs/wk 2. How often did you take breaks during practice sessions (please circle)?   Never Occasionally Quite frequently Very frequently   1. How long was each break, on average? (minutes) .................. 2. How long did you practise before you took each break? ...............................mins 3. How often did you warm up ON your instrument (e.g. slow scales, long tones, finger exercises) beefore practising or playing (please circle)?   Never Occasionally Quite frequently Very frequently   1. How often did you warm up AWAY from your instrument (e.g. movement, stretching, cardiovascular, core muscle exercises) before practising or playing (please circle)?   Never Occasionally Quite frequently Very frequently |
| 1. During the course of your training, have you received advice on the following (please tick)?  \|  \| **Yes** \| **No** \| \| --- \| --- \| --- \| \| **How to sit comfortably when playing (including correct chair height, type of chair, position of stand)** \|  \|  \| \| **How to play with flexibility and free movement** \|  \|  \| \| **When to take breaks during playing and practice sessions** \|  \|  \| \| **How to pace yourself during periods of intensive practice and playing** \|  \|  \| \| **How to look after your muscles and prevent strain** \|  \|  \| \| **How to warm up (away from your instrument)** \|  \|  \| \| **How to warm up (on your instrument)** \|  \|  \| \| **Why you should engage in aerobic/cardio physical activity** \|  \|  \| \| **Why you should do muscle strengthening exercises** \|  \|  \| |
| Your physical activity  We are interested in your knowledge of the official guidelines on physical activity for health purposes. The more honest your responses, the more helpful for us!   1. Do you know what the national recommendations are for taking part in physical activity, in terms of minutes per week of moderate intensity physical activity? (Please circle)   Yes No   1. If yes, what are the national recommendations for taking part in physical activity, in terms of minutes per week of moderate intensity physical activity?   .................................... minutes/week   1. Do you know what the national recommendations are for taking part in MUSCLE STRENGTHENING exercises only (legs, hips, back, abdomen, chest, shoulders and arms), in terms of days per week? (Please circle)   Yes No   1. If yes, what are the national recommendations for taking part in MUSCLE STRENGTHENING exercises only (legs, hips, back, abdomen, chest, shoulders and arms), in terms of days per week?   .................................... days/week |
| We are interested in finding out about the kinds of physical activities that people do as part of their everyday lives. The questions will ask you about the time you spent being physically active in the last 7 days. Please answer each question even if you do not consider yourself to be an active person. Please think about the activities you do at work, as part of your house and yard work (gardening), to get from place to place, and in your spare time for recreation, exercise or sport.  Think about all the VIGOROUS activities that you did in the LAST 7 DAYS. VIGOROUS physical activities refer to activities that take hard physical effort and make you breathe much harder than normal. Think ONLY about those physical activities that you did for at least 10 minutes at a time.  During the LAST 7 DAYS, on how many days did you do VIGOROUS physical activities like heavy lifting, digging, aerobics, or fast bicycling?   1. Number of days per week (if none, skip to question 27) |
| 1. How much time in HOURS and MINUTES did you usually spend doing VIGOROUS physical activities in ONE of those days?   (Write your answer in the following format: HH:MM. Example: 00:17 - seventeen minutes) |
| Think about all the MODERATE activities that you did in the LAST 7 DAYS. MODERATE activities refer to activities that take moderate physical effort and make you breathe somewhat harder than normal. Think only about those physical activities that you did for at least 10 minutes at a time.  During the LAST 7 DAYS, on how many days did you do MODERATE physical activities like carrying light loads, bicycling at a regular pace, or doubles tennis? Do not include walking.   1. Number of days per week (if none, skip to question 29) |
| 1. How much time in HOURS and MINUTES did you usually spend doing MODERATE physical activities on ONE of those days?   (Write your answer in the following format: HH:MM. Example: 00:17 - seventeen minutes) |
| Think about the time you spent WALKING in the LAST 7 DAYS. This includes at work and at home, walking to travel from place to place, and any other walking that you might do solely for recreation, sport, exercise, or leisure.  During the LAST 7 DAYS, on how many days did you WALK for at least 10 minutes at a time?   1. Number of days per week (if none, skip to question 31) |
| 1. How much time in hours and minutes (HH:MM) did you usually spend WALKING on ONE of those days? (Write your answer in the following format: HH:MM. Example: 00:20 - twenty minutes) |
| 1. Please indicate the extent to which you agree with the statements below regarding your physical activity by circling the appropriate number.  \|  \| **1**  **Strongly Disagree** \| **2** \| **3** \| **4** \| **5** \| **6** \| **7**  **Strongly Agree** \| \| --- \| --- \| --- \| --- \| --- \| --- \| --- \| --- \| \| I know what the recommended levels of physical activity are \| 1 \| 2 \| 3 \| 4 \| 5 \| 6 \| 7 \| \| I DO NOT know the reasons why I should be meeting the nationally recommended physical activity guidelines \| 1 \| 2 \| 3 \| 4 \| 5 \| 6 \| 7 \| \| I have NOT previously read information about the current nationally recommended physical activity guidelines \| 1 \| 2 \| 3 \| 4 \| 5 \| 6 \| 7 \| \| Facilities are available to help me to do physical activity \| 1 \| 2 \| 3 \| 4 \| 5 \| 6 \| 7 \| \| There is NOWHERE to do physical activity near me \| 1 \| 2 \| 3 \| 4 \| 5 \| 6 \| 7 \| \| My local area is NOT very attractive and this puts me off doing physical activity \| 1 \| 2 \| 3 \| 4 \| 5 \| 6 \| 7 \| \| I want to do physical activity \| 1 \| 2 \| 3 \| 4 \| 5 \| 6 \| 7 \| \| I CANNOT be bothered to do physical activity \| 1 \| 2 \| 3 \| 4 \| 5 \| 6 \| 7 \| \| I feel motivated to do physical activity \| 1 \| 2 \| 3 \| 4 \| 5 \| 6 \| 7 \| \| I DO NOT feel confident when doing physical activity \| 1 \| 2 \| 3 \| 4 \| 5 \| 6 \| 7 \| \| Doing physical activity makes me feel embarrassed \| 1 \| 2 \| 3 \| 4 \| 5 \| 6 \| 7 \| \| I FIND IT HARD to do physical activity when I see others doing well at physical activity (e.g. watching others run for a long time on the treadmill) \| 1 \| 2 \| 3 \| 4 \| 5 \| 6 \| 7 \| \| I can do physical activity to a good enough standard \| 1 \| 2 \| 3 \| 4 \| 5 \| 6 \| 7 \| \| I’ve NEVER really had sports skills so I DON’T do physical activity \| 1 \| 2 \| 3 \| 4 \| 5 \| 6 \| 7 \| \| I don’t seem to have the skills to keep going in physical activity sessions \| 1 \| 2 \| 3 \| 4 \| 5 \| 6 \| 7 \| \| Daily life is too stressful for physical activity \| 1 \| 2 \| 3 \| 4 \| 5 \| 6 \| 7 \| \| I have too many negative emotions which prevent me from doing physical activity \| 1 \| 2 \| 3 \| 4 \| 5 \| 6 \| 7 \| \| When I think about doing physical activity, I start to worry \| 1 \| 2 \| 3 \| 4 \| 5 \| 6 \| 7 \| \| My friends DON’T support or encourage my physical activity \| 1 \| 2 \| 3 \| 4 \| 5 \| 6 \| 7 \| \| The people I spend my free time with don’t do physical activity \| 1 \| 2 \| 3 \| 4 \| 5 \| 6 \| 7 \| \| I DON’T have anyone to do physical activity with \| 1 \| 2 \| 3 \| 4 \| 5 \| 6 \| 7 \| \| If I do physical activity, it will benefit me in the short term (e.g. burn calories, sleep better etc.) \| 1 \| 2 \| 3 \| 4 \| 5 \| 6 \| 7 \| \| If I do physical activity it will benefit me in the long term (e.g. live longer, lose weight etc.) \| 1 \| 2 \| 3 \| 4 \| 5 \| 6 \| 7 \| \| I think physical activity will change my life for the better \| 1 \| 2 \| 3 \| 4 \| 5 \| 6 \| 7 \| \| I tend to plan where my physical activity will happen (e.g. at the park, leisure centre etc.) \| 1 \| 2 \| 3 \| 4 \| 5 \| 6 \| 7 \| \| I do not tend to plan when my physical activity will happen (e.g. Monday at 6pm etc.) \| 1 \| 2 \| 3 \| 4 \| 5 \| 6 \| 7 \| \| I tend to plan how my physical activity will happen (e.g. how to get there, kit needed etc.) \| 1 \| 2 \| 3 \| 4 \| 5 \| 6 \| 7 \| \| I do not tend to plan what type of physical activity I will do (e.g. aerobics class, walking to work, session at the gym etc.) \| 1 \| 2 \| 3 \| 4 \| 5 \| 6 \| 7 \| \| I know what to do in difficult situations in order to make sure I do the physical activity I have planned \| 1 \| 2 \| 3 \| 4 \| 5 \| 6 \| 7 \| \| I get easily distracted from the physical activity I have planned \| 1 \| 2 \| 3 \| 4 \| 5 \| 6 \| 7 \| \| I always work around obstacles to physical activity; nothing really stops me \| 1 \| 2 \| 3 \| 4 \| 5 \| 6 \| 7 \| \| I WOULD NOT be prepared to give up work/practice ambitions to do physical activity \| 1 \| 2 \| 3 \| 4 \| 5 \| 6 \| 7 \| \| I would be prepared to give up things I usually do in my leisure time for physical activity \| 1 \| 2 \| 3 \| 4 \| 5 \| 6 \| 7 \| \| I WOULD NOT be prepared to give up spending time with my friends for physical activity \| 1 \| 2 \| 3 \| 4 \| 5 \| 6 \| 7 \| |
| In each of the past 4 WEEKS (i.e. over the past month), how many times did you do physical activities or exercises to STRENGTHEN your muscles? Do NOT count the times you did aerobic activities such as walking, running, or bicycling. Count the times you did activities using your own body weight such as yoga, sit-ups or push-ups and those using weight machines, free weights, or elastic bands. If none, skip to question 34.   1. ...............times per week 2. ...............times per month |
| 1. Listed below are some of the reasons that people give for not getting as much physical activity as they think they should. Please read each statement and indicate how likely you would be to give each of the following reasons:  \|  \| **Very unlikely**  **0** \| **Somewhat unlikely**  **1** \| **Somewhat likely**  **2** \| **Very likely**  **3** \| \| --- \| --- \| --- \| --- \| --- \| \| My day is so busy now, I just don’t think I can make the time to include physical activity in my regular schedule. \|  \|  \|  \|  \| \| None of my family members or friends like to do anything active, so I don’t have a chance to exercise. \|  \|  \|  \|  \| \| I’m just too tired after studying and practicing to get any exercise. \|  \|  \|  \|  \| \| I’ve been thinking about getting more exercise, but I just can’t seem to get started. \|  \|  \|  \|  \| \| I’m getting older so exercise can be risky. \|  \|  \|  \|  \| \| I don’t get enough exercise because I have never learned the skills for any sport. \|  \|  \|  \|  \| \| I don’t have acccess to jogging trails, swimming pools, bike paths, etc. \|  \|  \|  \|  \| \| Physical activity takes too much time away from other commitments – time, practice, study, family, etc. \|  \|  \|  \|  \| \| I’m embarrassed about how I will look when I exercise with others. \|  \|  \|  \|  \| \| I don’t get enough sleep as it is. I just couldn’t get up early or stay up late to get some exercise. \|  \|  \|  \|  \| \| It’s easier for me to find excuses not to exercise than to go out to do something. \|  \|  \|  \|  \| \| I know of too many people who have hurt themselves by overdoing it with exercise. \|  \|  \|  \|  \| \| I really can’t see learning a new sport at my age. \|  \|  \|  \|  \| \| It’s just too expensive. You have to take a class or join a club or buy the right equipment. \|  \|  \|  \|  \| \| My free times during the day are too short to include exercise. \|  \|  \|  \|  \| \| My usual social activities with family or friends do not include physical activity. \|  \|  \|  \|  \| \| I’m too tired during the week and I need the weekend to catch up on my rest. \|  \|  \|  \|  \| \| I want to get more exercise, but I just can’t seem to make myself stick to anything. \|  \|  \|  \|  \| \| I’m afraid I might injure myself or have a heart attack. \|  \|  \|  \|  \| \| I’m not good enough at any physical activity to make it fun. \|  \|  \|  \|  \| \| If we had exercise facilities and showers in college, then I would be more likely to exercise. \|  \|  \|  \|  \| |
| Your sedentary behaviour  Sedentary behaviour refers to any waking activity characterized by a reduced energy expenditure *and*a sitting or reclining posture.  In general, this means that any time a person is sitting or lying down, they are engaging in sedentary behaviour. Common sedentary behaviours include TV viewing, video game playing, computer use (collectively termed “screen time”), driving automobiles, and reading. |
| 1. On a typical WEEKDAY, how much time do you spend (from when you wake up until you go to bed) doing the following? (Please tick)  \|  \| **None** \| **15 min. or less** \| **30 min.** \| **1 hr** \| **2 hrs** \| **3 hrs** \| **4 hrs** \| **5 hrs** \| **6 hrs or more** \| \| --- \| --- \| --- \| --- \| --- \| --- \| --- \| --- \| --- \| --- \| \| Watching TV (including videos on VCR/DVD/computer). \|  \|  \|  \|  \|  \|  \|  \|  \|  \| \| Playing computer or video games. \|  \|  \|  \|  \|  \|  \|  \|  \|  \| \| Sitting listening to music on the radio, tapes, or CDs. \|  \|  \|  \|  \|  \|  \|  \|  \|  \| \| Sitting and talking on the phone. \|  \|  \|  \|  \|  \|  \|  \|  \|  \| \| Doing paperwork or computer work (office work, emails, paying bills, etc.) \|  \|  \|  \|  \|  \|  \|  \|  \|  \| \| Sitting reading a book or magazine. \|  \|  \|  \|  \|  \|  \|  \|  \|  \| \| Playing a musical instrument. \|  \|  \|  \|  \|  \|  \|  \|  \|  \| \| Doing artwork or crafts. \|  \|  \|  \|  \|  \|  \|  \|  \|  \| \| Sitting and driving in a car, bus, or train. \|  \|  \|  \|  \|  \|  \|  \|  \|  \| |
| 1. On a typical WEEKEND DAY, how much time do you spend (from when you wake up until you go to bed) doing the following? (Please tick)  \|  \| **None** \| **15 min. or less** \| **30 min** \| **1 hr** \| **2 hrs** \| **3 hrs** \| **4 hrs** \| **5 hrs** \| **6 hrs or more** \| \| --- \| --- \| --- \| --- \| --- \| --- \| --- \| --- \| --- \| --- \| \| Watching TV (including videos on VCR/DVD/computer). \|  \|  \|  \|  \|  \|  \|  \|  \|  \| \| Playing computer or video games. \|  \|  \|  \|  \|  \|  \|  \|  \|  \| \| Sitting listening to music on the radio, tapes, or CDs. \|  \|  \|  \|  \|  \|  \|  \|  \|  \| \| Sitting and talking on the phone. \|  \|  \|  \|  \|  \|  \|  \|  \|  \| \| Doing paperwork or computer work (office work, emails, paying bills, etc.) \|  \|  \|  \|  \|  \|  \|  \|  \|  \| \| Sitting reading a book or magazine. \|  \|  \|  \|  \|  \|  \|  \|  \|  \| \| Playing a musical instrument. \|  \|  \|  \|  \|  \|  \|  \|  \|  \| \| Doing artwork or crafts. \|  \|  \|  \|  \|  \|  \|  \|  \|  \| \| Sitting and driving in a car, bus, or train. \|  \|  \|  \|  \|  \|  \|  \|  \|  \| |
| Pain   1. How much bodily pain have you had during the past 4 weeks? (Please tick)   ❑ None  ❑ Very mild  ❑ Mild  ❑ Moderate  ❑ Severe  ❑ Very severe   1. During the past 4 weeks, how much did pain interfere with your practice and performance? (Please tick)   ❑ Not at all  ❑ A little bit  ❑ Moderately  ❑ Quite a bit  ❑ Extremely |
| Performance-related musculoskeletal disorders (PRMDs)  Performance related musculoskeletal disorders (PRMDs) are defined as any pain, weakness, numbness, tingling or any other symptoms that interfere with your ability to play your instrument at the level you are accustomed to. This definition does NOT include mild transient aches or pains. |
| 1. Have you ever been given any information on how to prevent playing-related pain (please circle)?   Yes No   1. If yes, from what sources (e.g. magazine, doctor, teacher, lecture-demonstration, health care practitioner)?   ........................................................................................................................................................................... |
| 1. In general, have you found it easy to access information about preventing/treating PRMDs (please circle)?   Yes No |
| 1. Please indicate how often you suffer from a PRMD by circling the most appropriate number:   Never 0 1 2 3 4 5 6 7 8 9 10 Constantly   1. Please indicate the average severity of any PRMD that you suffer from:   None 0 1 2 3 4 5 6 7 8 9 10 Maximally severe |
| A Rating of Physical Exertion (RPE) represents the amount of effort needed to carry out an activity on a scale of 6 to 20 in which 6 represents no effort, 13 represents “somewhat hard at the time”, and 20 represents maximal effort.  Please answer the following questions with reference to the past 7 DAYS. (If the past 7 days represent do NOT represent a typical week, please think back to the most recent typical week.)   1. Please circle the number below that you feel best represents the amount of effort you needed to carry out your daily practice:   RPE SCALE  6  7 very, very light  8  9 very light  10  11 fairly light  12  13 somewhat hard  14  15 hard  16  17 very hard  18  19 very, very hard  20 |
|  |

| **Anxiety**  **Tick the box beside the reply that is closest to how you have been feeling over the past 7 DAYS. Please don’t take too long over your replies: we want to know your immediate response to each item.**   \|  \| Most of the time \| A lot of the time \| From time to time, occasionally \| Not at all \| \| --- \| --- \| --- \| --- \| --- \| \| 1. **I feel tense or ‘wound up’** \|  \|  \|  \|  \|  \|  \| Very definitely and quite badly \| Yes, but not too badly \| A little, but it doesn’t worry me \| Not at all \| \| --- \| --- \| --- \| --- \| --- \| \| 1. **I get a sort of frightened feeling as if something awful is about to happen** \|  \|  \|  \|  \| |
| --- | --- | --- | --- | --- | --- | --- | --- | --- | --- | --- | --- | --- | --- | --- | --- | --- | --- | --- | --- | --- |
| \|  \| A great deal of the time \| A lot of the time \| From time to time, but not too often \| Only occasionally \| \| --- \| --- \| --- \| --- \| --- \| \| 1. **Worrying thoughts go through my mind** \|  \|  \|  \|  \|  \|  \| Definitely \| Usually \| Not often \| Not at all \| \| --- \| --- \| --- \| --- \| --- \| \| 1. **I can sit at ease and feel relaxed** \|  \|  \|  \|  \|  \|  \| Not at all \| Occasionally \| Quite often \| Very often \| \| --- \| --- \| --- \| --- \| --- \| \| 1. **I get a sort of frightened feeling like ‘butterflies’ in the stomach** \|  \|  \|  \|  \|  \|  \| Very much indeed \| Quite a lot \| Not very much \| Not at all \| \| --- \| --- \| --- \| --- \| --- \| \| 1. **I feel restless as I have to be on the move** \|  \|  \|  \|  \|  \|  \| Very often indeed \| Quite often \| Not very often \| Not at all \| \| --- \| --- \| --- \| --- \| --- \| \| 1. **I get sudden feelings of panic** \|  \|  \|  \|  \| |
| Thank you very much for completing this questionnaire!  If any issues have arisen for you as a result of completing this questionnaire, please contact the British Association for Performing Arts Medicine (BAPAM), via telephone (020 7404 8444) or email [info@bapam.org.uk](mailto:info@bapam.org.uk) |
